# Rapid Colorimetric Detection of Cartap Residues by AgNP Sensor with Magnetic Molecularly Imprinted Microspheres as Recognition Elements

**DOI:** 10.3390/molecules23061443

**Published:** 2018-06-14

**Authors:** Mao Wu, Huiyun Deng, Yajun Fan, Yunchu Hu, Yaping Guo, Lianwu Xie

**Affiliations:** 1 College of Sciences, Central South University of Forestry and Technology, Changsha 410004, China; wumaoweiming@126.com (M.W.); denghuiyuncsuft@126.com (H.D.); fanyajuncsfu@126.com (Y.F.); huyunchu@csuft.edu.cn (Y.H.); 2 College of Chemistry and Chemical Engineering, Central South University, Changsha 410083, China

**Keywords:** cartap residue, colorimetric determination, tea beverage, molecularly imprinted microsphere, selective recognition, silver nanoparticle sensor

## Abstract

The overuse of cartap in tea tree leads to hazardous residues threatening human health. A colorimetric determination was established to detect cartap residues in tea beverages by silver nanoparticles (AgNP) sensor with magnetic molecularly imprinted polymeric microspheres (Fe_3_O_4_@mSiO_2_@MIPs) as recognition elements. Using Fe_3_O_4_ as supporting core, mesoporous SiO_2_ as intermediate shell, methylacrylic acid as functional monomer, and cartap as template, Fe_3_O_4_@mSiO_2_@MIPs were prepared to selectively and magnetically separate cartap from tea solution before colorimetric determination by AgNP sensors. The core-shell Fe_3_O_4_@mSiO_2_@MIPs were also characterized by FT-IR, TEM, VSM, and experimental adsorption. The Fe_3_O_4_@mSiO_2_@MIPs could be rapidly separated by an external magnet in 10 s with good reusability (maintained 95.2% through 10 cycles). The adsorption process of cartap on Fe_3_O_4_@mSiO_2_@MIPs conformed to Langmuir adsorption isotherm with maximum adsorption capacity at 0.257 mmol/g and short equilibrium time of 30 min at 298 K. The AgNP colorimetric method semi-quantified cartap ≥5 mg/L by naked eye and quantified cartap 0.1–5 mg/L with LOD 0.01 mg/L by UV-vis spectroscopy. The AgNP colorimetric detection after pretreatment with Fe_3_O_4_@mSiO_2_@MIPs could be successfully utilized to recognize and detect cartap residues in tea beverages.

## 1. Introduction

Cartap, an analogue of the natural insecticide nereistoxin, has been widely used to kill destructive pests in rice, vegetables, fruit trees and tea trees all over the world [1,2]. In spite of its low mammalian toxicity, the overuse of cartap could lead to hazardous residues threatening human health [3]. Hence, maximum residue limits for cartap have been ascertained by the food administrations of many nations including China. For example, the Ministry of Agriculture of China had set the maximum residue limit of cartap at 20 mg/kg for tea [4].

Aiming to reduce the risk of polluting the environment and to establish a safety standard of cartap, determining cartap content in food is of great importance. Various analytical methods have been used for detecting cartap, such as spectrophotometry [3,5,6], thin-layer chromatography [7], gas chromatography [8], high-performance liquid chromatography [9,10], gas chromatography-mass spectrometry [11,12,13], liquid chromatography-tandem mass spectrometry [14]. These methods are successfully applied to qualitatively and quantitatively determine cartap. However, most of operation procedures of the above analytical methods are not only laborious and time-consuming, but also require expensive instruments and many organic solvents. In addition, in some cases, cartap must be transformed into nereistoxin compounds under alkaline conditions before detection [15]. Thus, novel, low-cost, rapid, concise, and accurate analytical methods such as colorimetric analysis are still in significant demand.

Silver nanoparticles (AgNP)-based colorimetric analysis had been paid more attention in recent years. AgNPs present intense colors which will produce characteristic absorption peaks due to the effect of surface plasmon resonance (SPR) [16]. The SPR effect derives from the size and shape of particles, inter-particle distances, and the dielectric properties of surroundings [17] which are related to the aggregation of nanoparticles. Extra substances added into the AgNP system can cause the inter-particle distance to change and subsequently result in quantifiable shifts of the UV–vis absorption, which is the fundamental principle of the colorimetric sensor [18]. Owing to the rapid, sensitive, and easy-to-use characteristics, AgNP colorimetric detection methods have been widely investigated to identify many chemical and biological molecules [19,20,21,22]. Nevertheless, the poor selectivity of those colorimetric methods has restricted its application in complicated sample matrices (e.g., foods) because copious interferents may also lead to AgNP aggregation. Because of that, numerous measures were taken to improve the selectivity of AgNP colorimetric sensors. For instance, Yuan et al. used amine-terminated generation 5 poly (amidoamine) dendrimers-stabilized AgNPs to calorimetrically detect mercury ions in aqueous solution [23]. Patel et al. developed a colorimetric method for the sensitive and selective detection of carbendazim fungicide in water and food samples using 4-aminobenzenethiol functionalized AgNPs as a colorimetric sensor [24]. However, synthesizing these AgNPs is complex and the sensitivity may be influenced by the stabilizers due to their prohibiting effects on the aggregation of AgNPs. Therefore, separation and enrichment of analyte from sample matrices need to be carried out properly before the analysis.

Generally, sample pretreatments using liquid–liquid extraction, precipitation, and centrifugation are largely employed before applying the above-mentioned colorimetric sensing techniques. For example, Liu et al. reported a visible spectrophotometry for the rapid assay of cartap with citrate-coated Au nanoparticles after the sample was pretreated by multiple liquid–liquid extractions, and cartap could be detected in the range of 0.05–0.6 mg/kg [3]. However, the pretreatments lack the selectivity and cannot completely remove the interferents. Other methods such as antibody-based [25] and aptamer-based [26] separation methods have been successfully established to separate and enrich pesticide residues from complex foodstuffs. However, high cost and laborious manufacture of antibody and aptamer critically restrict their application in conventional detection of pesticide residues in foods.

Lately, molecularly imprinted polymers (MIPs)-based pretreatment methods have been developed to successfully enrich some metabolites in vivo [27], food addictive Sudan [28], triazine herbicides [29], chlorpyrifos [18], trichlorfon [30], and organophosphorus pesticide residues [31] in diverse food products. In particular, core-shell magnetic MIPs with magnetic Fe_3_O_4_ as support (Fe_3_O_4_@mSiO_2_@MIPs) have been considered as desirable and have received increasing attention [32,33] owing to some notable advantages of unique magnetic properties for easily and rapidly recognizing and separating analytes from the matrix by external magnets after adsorption without a column-packing procedure [34,35,36,37,38]. Up to now, there has been no report involving the preparation of Fe_3_O_4_@mSiO_2_@MIPs with cartap as a template to efficaciously and selectively isolate and enrich cartap from complicated matrices.

**Figure 1:**
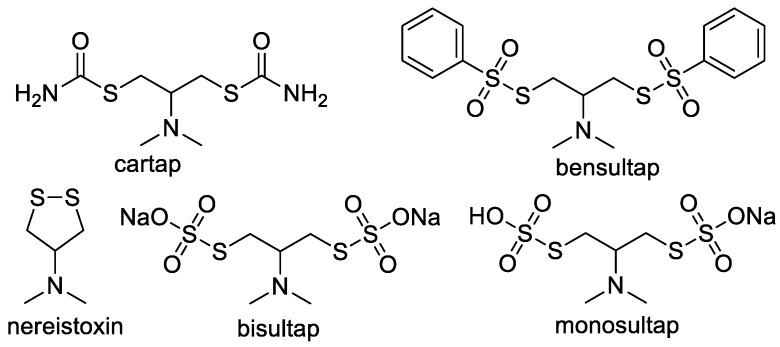
Chemical structures of cartap and its analogues.

The current research is conducted to develop a pretreatment with Fe_3_O_4_@mSiO_2_@MIPs as recognition elements before AgNP sensor colorimetric determination of cartap in tea beverages. Fe_3_O_4_@mSiO_2_@MIPs are prepared to specifically recognize cartap from mixture solution containing cartap and its four analogues including nereistoxin, bensultap, bisultap and monosultap (Figure 1). The AgNP colorimetric methods are exploited to high-throughput screen and semi-quantify cartap in tea beverages. As far as we know, there is no study that uses AgNP colorimetric detection with magnetic surface molecularly imprinted polymeric microspheres as recognition elements for high-throughput screening and semi-quantification of cartap in agri-food products.

## 2. Results and Discussion

### 2.1. Preparation of Fe_3_O_4_@mSiO_2_@MIPs

**Figure 2:**
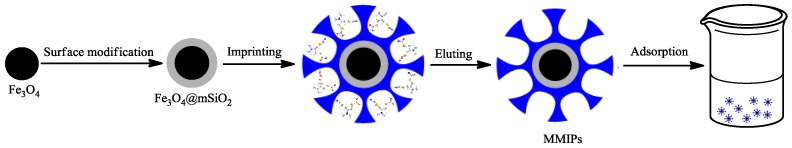
Schematics for the synthesis of Fe_3_O_4_@mSiO_2_@MIPs.

The preparation process of Fe_3_O_4_@mSiO_2_@MIPs is schematically shown in Figure 2. First, a solvothermal reaction was used to synthesize Fe_3_O_4_ microspheres which have higher magnetic response than those prepared by coprecipitation method. Then, a layer of cetyltrimethyl ammonium bromide (CTAB)/SiO_2_ composites was coated over the Fe_3_O_4_ microspheres through a concise one-step sol-gel process using CTAB as a template for forming mesoporous structure. After removing CTAB in a mild way by acetone soxhlet extraction, well-dispersed Fe_3_O_4_@mSiO_2_ particles with magnetic core and mesoporous silica shell were obtained [36]. By virtue of a higher ratio of surface to volume, mesoporous materials have remarkable advantages for preparing surface MIPs. IN particular, the mesoporous structure of silica shell could provide more surface area to graft various functional groups, which was beneficial to the adsorption of target molecules [39]. Besides, coating SiO_2_ over Fe_3_O_4_ would improve their dispersion in water, decrease agglomerations during reduplicated magnetic separation, and raise their reusability [40]. After that, in the presence of 2,2′-azobis(isobutyronitrile) (AIBN), vinyl groups were led into the surface of Fe_3_O_4_@mSiO_2_ with MPS for reaction with ethylene glycol dimethacrylate (EGDMA) to initiate the copolymerization with preassembly solution of methylacrylic acid (MAA) and cartap, which interacted mainly on the hydrogen bond. Ultimately, the surface binding sites of Fe_3_O_4_@mSiO_2_@MIPs were realized by removing the templates. Thus, a filmy layer of MIPs was synthesized on the surface of solid support with micro size, which was beneficial for improving the mass transfer rate and completely removing the template [41].

### 2.2. Characterization of Fe_3_O_4_@mSiO_2_@MIPs

**Figure 3:**
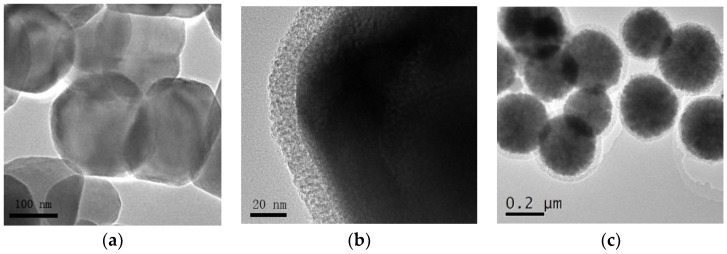
TEM images of (**a**) Fe_3_O_4_; (**b**) Fe_3_O_4_@mSiO_2_; and (**c**) Fe_3_O_4_@mSiO_2_@MIPs.

Morphology and particle size of prepared Fe_3_O_4_ nanoparticles, Fe_3_O_4_@mSiO_2_ and Fe_3_O_4_@mSiO_2_@MIPs can be observed by TEM (Figure 3). About 20 nm thick mSiO_2_ shell (Figure 3b) was uniformly coated onto Fe_3_O_4_ dark core with about 200 nm diameter (Figure 3a). After the process of surface imprinting polymerization, an external polymer layer with 50 nm diameter was distinctly found to be around Fe_3_O_4_@mSiO_2_ nanoparticles (Figure 3c), which indicated that the surface of Fe_3_O_4_@mSiO_2_ nanoparticles had been successfully grafted by the MIPs layer. Thus, the core-shell structural Fe_3_O_4_@mSiO_2_@MIPs with diameter of about 250 nm were successfully synthesized with regular spherical shape and relatively narrow size distribution (Figure 3c). As the cylindrical mesoporous in mSiO_2_ layer were perpendicular to the Fe_3_O_4_ surface (Figure 3b), the imprinting precursor penetrated into the channels and the MIPs layer could be riveted into the internal surface of Fe_3_O_4_@mSiO_2_ [42], which was favorable to more recognition sites and more strong capacity of adsorption.

In FT-IR spectra, the strong Fe-O vibration absorption peak at 580 cm^−1^ (Figure S1a), the Si-O symmetric stretching vibration peak at 800 cm^−1^, and strong Si-O-Si asymmetric stretching vibration peak at 1072 cm^−1^ (Figure S1b) indicated that SiO_2_ layer was successfully encapsulated on the surface of Fe_3_O_4_ nanoparticles. The characteristic absorption peaks at 2922 cm^−1^ and 2851 cm^−1^ (C-H stretching vibration) were ascribed to CTAB (Figure S1b). On the contrary, the inexistence of above C-H stretching vibration peaks in Figure S1c suggested the complete removal of CTAB. Moreover, the stretching vibration peak of the C=C bonds at 1632 cm^−1^ was ascribed to the successful modification with vinyl groups (Figure S1c). The adsorption peak of C=O stretching band at 1720 cm^−1^ (Figure S1d) for EDGMA indicated that the MIPs was successfully coated over the surface of Fe_3_O_4_@mSiO_2_.

**Figure 4:**
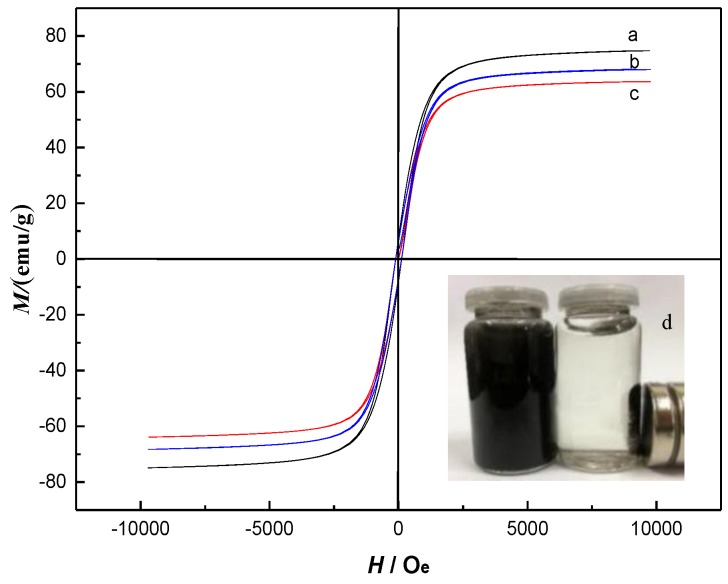
The magnetization curves of (**a**) Fe_3_O_4_; (**b**) Fe_3_O_4_@mSiO_2_; (**c**) Fe_3_O_4_@mSiO_2_@MIPs; and (**d**) the magnetic separation of Fe_3_O_4_@mSiO_2_@MIPs under the external magnet.

The magnetization curves of Fe_3_O_4_ nanoparticles, Fe_3_O_4_@mSiO_2_ and Fe_3_O_4_@mSiO_2_@MIPs are shown in Figure 4. The shape of the three curves is similar, there is no residual magnetism, and the value of coercive force is zero, which shows that three types of materials are superparamagnetic. The magnetic saturation of resultant Fe_3_O_4_@mSiO_2_@MIPs was approximately 66 emu/g (Figure 4c), which was lower than those of Fe_3_O_4_ nanoparticles (75 emu/g) and Fe_3_O_4_@mSiO_2_ (68 emu/g) (Figure 4a,b), which could be relevant to the magnetic inactive layer including mSiO_2_ and imprinted polymer layers. Nevertheless, the reduction of magnetism did not severely influence the magnetic separation of Fe_3_O_4_@mSiO_2_@MIPs, and they kept intensely magnetic and could be collected within 10 s in solution by an external magnet (Figure 4d) and scattered rapidly after a gentle shake once the magnetic field was withdrawn.

### 2.3. Adsorption Characteristics

Adsorption isotherms of cartap on Fe_3_O_4_@mSiO_2_@MIPs and magnetic molecularly imprinted microspheres in the absences of template (Fe_3_O_4_@mSiO_2_@NIPs) were determined at 298 K, 308 K and 318 K, respectively (Figure S2). The equilibrium adsorption capacity of cartap on Fe_3_O_4_@mSiO_2_@MIPs and Fe_3_O_4_@mSiO_2_@NIPs raised rapidly first, then lightly along with raising initial concentrations, and then approached saturation when the initial concentration reached 15.0 mmol/L. The equilibrium adsorption capacity of cartap on Fe_3_O_4_@mSiO_2_@MIPs at 298 K was 0.257 mmol/g, 9.5 times of that on Fe_3_O_4_@mSiO_2_@NIPs (0.027 mmol/g), which could be caused by the imprinting effect. Hence, Fe_3_O_4_@mSiO_2_@MIPs showed higher adsorption capacity to cartap and might be suitable for isolating cartap from complex samples.

In addition, the equilibrium adsorption capacity of cartap on two sorbents increased along with the increase of temperature, which is consistent with previous research results that MIPs synthesized at higher temperatures tend to work better owing to the similar 3D structures at similarly higher temperatures. Furthermore, at higher temperatures the solvent possesses a lower viscosity and surface tension and meanwhile can improve the wetting of the Fe_3_O_4_@mSiO_2_@MIPs, which then result in higher binding capacity to some extent. To estimate the binding performances of Fe_3_O_4_@mSiO_2_@MIPs or Fe_3_O_4_@mSiO_2_@NIPs further, two representatively isothermal models, namely the Langmuir Equation (1) and Freundlich Equation (2), were applied to fit the experimental data.

(1) $$\mathrm{Langmuir}\;\mathrm{equation}:\;\frac{1}{Q_{e}}=\frac{1}{Q_{m}K_{L}c_{e}}+\frac{1}{Q_{m}}$$

(2) $$\mathrm{Freundlich}\;\mathrm{equation}:\;\ln Q_{e}=\frac{1}{n}\ln c_{e}+\ln K_{F}$$

where *Q*_e_ and *Q*_m_ are the equilibrium adsorption capacity (mmol/g) and the maximum adsorption capacity (mmol/g) respectively, *c*_e_ (mmol/L) is the equilibrium concentration of cartap, *K*_L_ is a characteristic constant (L/mmol), *n* and *K*_F_ are Freundlich constants.

The Langmuir equation can be used for characterizing a monolayer adsorption, and the Freundlich equation can be used for characterizing a multilayer adsorption and a monolayer adsorption. Supplementary material Table S1 summarizes the fitted parameters *Q*_m_, *K*_L_, *K*_F_, *n* and R^2^ (correlation coefficient), which indicates that the Langmuir equation with R^2^ > 0.99 might be better fit the isotherm experimental data. In addition, *Q*_m_(cal) calculated by the Langmuir equation was closer to *Q*_m_(exp) measured in the experiment. Therefore, the adsorption process of cartap could be regarded as a monolayer adsorption. Moreover, the *Q*_m_(exp) of Fe_3_O_4_@mSiO_2_@MIPs of cartap at diverse temperatures were enormously higher than those of Fe_3_O_4_@mSiO_2_@NIPs, which demonstrated that Fe_3_O_4_@mSiO_2_@MIPs exhibited higher binding affinity for cartap than Fe_3_O_4_@mSiO_2_@NIPs.

Adsorption kinetic curves of adsorption of cartap on Fe_3_O_4_@mSiO_2_@MIPs and Fe_3_O_4_@mSiO_2_@NIPs at 318 K are shown in Figure S3. We found that the adsorption capacities of cartap raised with the adsorption time increasing. When the initial concentration of cartap was set at 4.0 mmol/L, the adsorption equilibrium of cartap (15 mL) on 50 mg Fe_3_O_4_@mSiO_2_@NIPs could be achieved in 15 min at 318 K. However, it took more time for Fe_3_O_4_@mSiO_2_@MIPs to attain adsorption equilibrium than Fe_3_O_4_@mSiO_2_@NIPs, which might be ascribed to the specific molecular recognition behavior on customized binding sites and stereo-cavity of Fe_3_O_4_@mSiO_2_@MIPs, while there was physical adsorption onto randomly distributed functional groups on the surface of Fe_3_O_4_@mSiO_2_@NIPs. The adsorption equilibrium time of adsorption of cartap on Fe_3_O_4_@mSiO_2_@MIPs was 30 min, which means that 30 min could be regarded as the longest extraction time. For non-surface imprinted polymer, achieving adsorption equilibrium generally needs 12–24 h [43]. Therefore, the binding sites of surface of microspheres provided by surface imprinting technology led to faster mass transfer for binding kinetics.

### 2.4. Evaluation of Selectivity for Recognition

**Figure 5:**
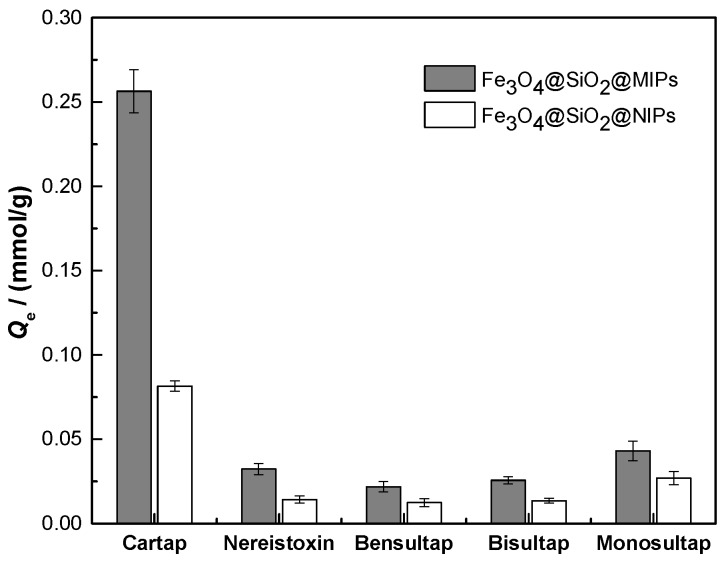
Adsorption capacities of cartap in the presence of its analogues (nereistoxin, bensultap, bisultap, monosultap) in mixture solution with the initial concentrations of 4.0 mmol/L for each one on Fe_3_O_4_@mSiO_2_@MIPs and Fe_3_O_4_@mSiO_2_@NIPs at 298 K.

Molecular recognition ability of Fe_3_O_4_@mSiO_2_@MIPs mainly depends on the binding between Fe_3_O_4_@mSiO_2_@MIPs and adsorbed molecules, and the binding ability is related to similarity between template and adsorbed molecules in functional groups, size and stereo structure [37]. Obviously, the adsorption capacities of five analogues including cartap, nereistoxin, bensultap, bisultap and monosultap (Figure 1) on Fe_3_O_4_@mSiO_2_@NIPs had no significant difference (Figure 5) because of non-specific adsorption. However, the adsorption capacities of five analogues on Fe_3_O_4_@mSiO_2_@MIPs were distinct from one and another and were much higher than those on Fe_3_O_4_@mSiO_2_@NIPs.

As shown in Figure 5, the imprinting factor *α* calculated for cartap on Fe_3_O_4_@mSiO_2_@MIPs was 3.1, which was much higher than those of *α* for nereistoxin (2.3), bensultap (1.8), bisultap (1.9) and monosultap (1.6) on Fe_3_O_4_@mSiO_2_@MIPs. The results demonstrated that Fe_3_O_4_@mSiO_2_@MIPs had specific adsorption to cartap and implied the success of imprinting process. When the initial concentration was set at 4.0 mmol/L for each one in mixed solution, the separation factors were calculated as *β*_cartap/nereistoxin_ = 7.9, *β*_cartap/bensultap_ = 11.8, *β*_cartap/bisultap_ = 10.0, *β*_cartap/monosultap_ = 5.9, the reason for which was that the specific sites were complementary in size, shape, and spatial distribution of cartap in Fe_3_O_4_@mSiO_2_@MIPs. Despite the same moiety of *N*,*N*-dimethyl-dithiolan-amine for five selected analogues, the adsorption capacity of cartap on Fe_3_O_4_@mSiO_2_@MIPs was higher than its analogues.

Four selected analogues had the same central skeleton of *N*,*N*-dimethyl-dithiolan-amine as cartap, but bensultap, bisultap, and monosultap had two sulfonic groups, respectively, and bensultap had another two hydrophobic benzene rings (Figure 1). The lower adsorption capacities for nereistoxin, bensultap, bisultap and monosultap were probably due to non-specific imprinted sites for compounds with different molecular size and stereochemistry [35]. For bensultap, the hydrophobic effect of the benzene rings probably kept it from getting into the binding cavities, and consequently, the binding capacity was weakened. Besides, for bisultap and monosultap, the superhydrophilicity and steric effect of the sulfonic group induced lower adsorption capacity. These results revealed that the imprinting effect of Fe_3_O_4_@mSiO_2_@MIPs could be significant for selective extraction of cartap from the real complicated matrix. Therefore, the properties of Fe_3_O_4_@mSiO_2_@MIPs were better than those traditional non-specific sorbents.

### 2.5. Reusability of Fe_3_O_4_@mSiO_2_@MIPs

The preliminary experimental results demonstrated that methanol-acetic acid (9/1, *v*/*v*) could attenuate the non-covalent interactions between target analytes and Fe_3_O_4_@mSiO_2_@MIPs, so that it was regarded as the optimal desorption solvent to remove templates to regenerate Fe_3_O_4_@mSiO_2_@MIPs. The reusability of Fe_3_O_4_@mSiO_2_@MIPs was researched by monitoring the adsorption efficiency of ten batch samples to extend its operational life span for lowering cost. After ten successive adsorption-desorption rounds, the adsorption efficiency was still as high as 95.2% of the original one (Figure S4), which demonstrates that Fe_3_O_4_@mSiO_2_@MIPs is relatively stable and could be reused ten times with almost no effect on its adsorption efficiency.

### 2.6. Selective Enrichment of Cartap from Spiked Tea Beverages

In this study, we applied a concise and rapid sample pretreatment process of solid phase extracting cartap from tea solution by Fe_3_O_4_@mSiO_2_@MIPs without cartridge packing. After absorbing the analytes in a vial, Fe_3_O_4_@mSiO_2_@MIPs/Fe_3_O_4_@mSiO_2_@NIPs were gathered by an external magnet, and then washed with acetonitrile and then with methanol-acetic acid (9/1, *v*/*v*) to release analytes [44].

Taking the recovery of cartap and handling time into account, we set the solid-to-liquid ratio of Fe_3_O_4_@mSiO_2_@MIPs/Fe_3_O_4_@mSiO_2_@NIPs to tea solution at 10:3 (mg:mL) and extraction time at 30 min based on the adsorption experiment. The total recoveries of cartap from tea solution after solid phase extraction were confirmed using HPLC. In general, the Fe_3_O_4_@mSiO_2_@MIPs extraction realized almost absolute recovery (>95%) of cartap from tea solution while approximately half of the cartap was lost after being treated by Fe_3_O_4_@mSiO_2_@NIPs (data not shown). Moreover, the recovery of cartap from the tea solution decreased with the increase in cartap concentration after being absorbed by both Fe_3_O_4_@mSiO_2_@MIPs and Fe_3_O_4_@mSiO_2_@NIPs. This could be result from the adsorption saturation and the decreased equilibrium rate of the polymers.

### 2.7. Colorimetric and UV-Vis Spectroscopic Determination of Cartap

**Figure 6:**
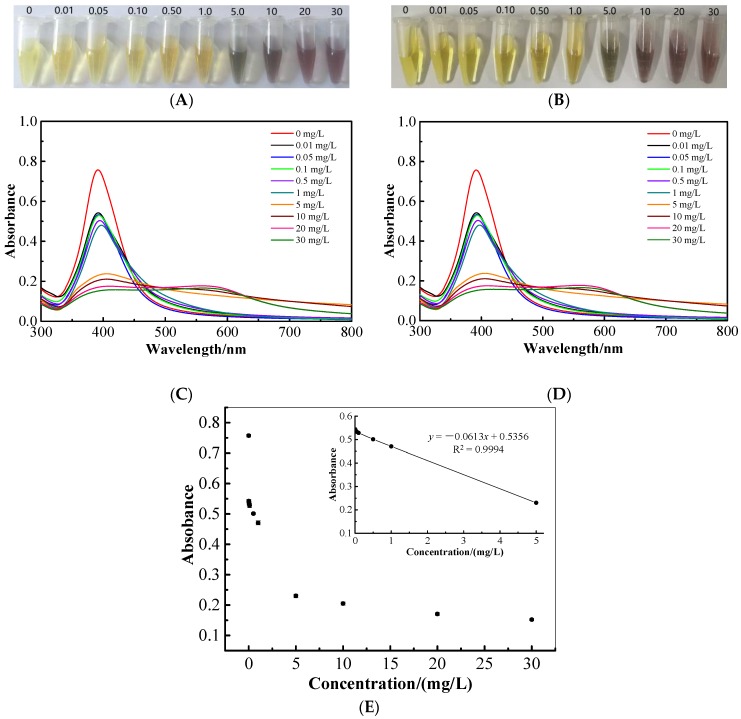
Representative photographic image of colorimetric sensing with (**A**) different concentrations (mg/L) of cartap standard solutions and with (**B**) Fe_3_O_4_@mSiO_2_@MIPs extracts of bottled green tea beverage spiked with different concentrations (mg/L) of cartap; average UV–vis absorption spectra of AgNPs (**C**) in the presence of different concentrations of cartap and (**D**) in the presence of Fe_3_O_4_@mSiO_2_@MIPs extracts of bottled green tea beverage spiked with different concentrations of cartap; (**E**) plot of the average absorption values at 392 nm versus different concentrations of cartap standard solutions (*n* = 3).

Due to the selectivity and effectivity of Fe_3_O_4_@mSiO_2_@MIPs for recognizing and separating cartap from tea beverages, there is no need to take further measurements to ensure the selectivity for the colorimetric determination. After adding cartap standard solutions into the freshly synthesized AgNP colloidal solutions, the changes of the solution’s color were recorded (Figure 6A). It illustrated that the color changed from yellow to brown with cartap concentrations raising from 0.01 to 5 mg/L, and then turned to light purple at 10 mg/L. The purple color of the solution diminished and came to be light gray when cartap concentration reached 40 mg/L due to the intensive aggregation of AgNPs. Hence, we could roughly mensurate the concentrations of cartap ≥5 mg/L by the naked eye based on the colors of the solutions. Nevertheless, when the concentration of cartap was ≤1 mg/L, there was no observable variation in color. To enhance the sensitivity of this colorimetric sensor, UV-vis spectra was scanned as showing in Figure 6C. The absorbance intensity at 392 nm decreased with the concentration of cartap raising and red shift of the spectra was observed at the same time. The plot of the absorbance of cartap with different concentrations at 392 nm versus the concentration is shown in Figure 6E. The linear regression model could not be testified to correlate the absorbance with cartap concentrations at the range of 0–30 mg/L. Anyway, an intense linear relationship (R^2^ = 0.9994) was determined at the cartap concentrations changing from 0.1 to 5 mg/L, revealing the practicability of using UV-vis spectral colorimetric methods to quantitate cartap of concentrations from 0.1 to 5 mg/L. Fortunately, this value could satisfy the maximum residue limit proposed by the Ministry of Agriculture of China for cartap in teas.

The corresponding tests of using Fe_3_O_4_@mSiO_2_@MIPs to pretreat samples prior to colorimetric sensor determination of cartap in spiked bottled green tea beverages were also conducted. The results shown in Figure 6B,D are consistent with the results of cartap standard solutions quite well, and the extraction recovery rate of 98.3–101.6% was identified by HPLC, implying the practicability of using AgNP-colorimetric sensors for the measurement of cartap residue in tea beverages.

Compared with AgNP colorimetric method after Fe_3_O_4_@mSiO_2_@MIPs pretreatment, the corresponding samples had been analyzed by HPLC at the same time, and the detection results by the two methods were extremely close to each other, and we found that there were no significant differences between the proposed method and traditional HPLC method (Table 1), so the colorimetric analysis method based on AgNP sensor with Fe_3_O_4_@mSiO_2_@MIPs as recognition elements could be used to detect the cartap in tea products or bottled tea beverages.

## 3. Materials and Methods

### 3.1. Chemicals and Reagents

Sodium acetate, FeCl_3_·6H_2_O, polyethylene glycol 6000 (PEG 6000), cetyltrimethyl ammonium bromide (CTAB), tetraethyl orthosilicate (TEOS), acetonitrile, ethanol, acetic acid, acetone and HPLC grade methanol were purchased from Sinopharm Chemical Reagent Co., Ltd. (Shanghai, China). Acrylamide (AM), acrylic acid (AA), methylacrylic acid (MAA), 4-vinylpyridine (4-VP), ethylene glycol dimethacrylate (EGDMA), and 3-(trimethoxysily) propyl methacrylate (MPS) were acquired from Shanghai Macklin Biochemical Co, Ltd. (Shanghai, China). 2,2′-azobis-(isobutyronitrile) (AIBN) was provided by Chinasun Specialty Products co, Ltd. (Jiangsu, China). Cartap with purities over 98% was purchased from RAM.M Reagent Company, China. Nereistoxin and bensultap with purities over 99% were obtained from Alfa Chemistry, New York, NY, USA. Both bisultap and monosultap with purities over 99% were from Crescent Chemical Company, Islandia, NY, USA. Triple distilled water (deionized water) newly collected from a glass distillator (Yarong, Shanghai, China) was used to prepare solutions. Other reagents were of analytical grade and obtained from Sinopharm Chemical Reagent Co., Ltd. (Shanghai, China). Six tea samples (green, black, white, yellow, dark and oolong teas), bottled green tea beverage, and bottled iced black tea beverage were purchased from a local supermarket.

### 3.2. Apparatus and Conditions

FT-IR spectra (4000–400 cm^−1^) of various particles were obtained from a Nicolet 6700 FT-IR spectrometer (Thermo Nicolet Co., Waltham, MA, USA). The morphology of microspheres was observed on a TEM (Tecnai G2 F20, FEI, Hillsboro, OR, USA). The magnetic property was identified using VSM (Squid-VSM, Quantum Design, San Diego, CA, USA) at room temperature. HPLC analysis was performed on a Purkinje L600 HPLC system (Beijing, China) equipped with a Pgrandsil-STC-C_18_ column (250 mm × 4.6 mm, 5 μm, Bonna-Agela, Wilmington, DE, USA) and a UV spectrum detector. With methanol- water (*v*/*v* = 20:80) mixture containing 0.17% acetic acid as the mobile phase, the chromatogram was acquired at 238 nm with the rate of flow 0.5 mL/min and the column temperature 25 °C. UV-vis spectra were scanned on an UV2800 UV-vis spectrophotometry (Sunny Hengping Scientific Instrument Co, Ltd., Shanghai, China).

### 3.3. Preparation of Fe_3_O_4_@mSiO_2_@MIPs

Fe_3_O_4_@mSiO_2_@MIPs with cartap as template were synthesized by a surface-imprinted polymerization method using Fe_3_O_4_ nanoparticles as support according to our previous work [35] with minor modifications as showing in Figure 2. Firstly, magnetic Fe_3_O_4_ nanoparticles, Fe_3_O_4_@mSiO_2_ microspheres, and vinyl-modified Fe_3_O_4_@mSiO_2_ microspheres were prepared in turn according to our previously published method [35,37].

Then, 0.25 mmol cartap and 1.0 mmol MAA were dissolved in 6.0 mL anhydrous acetonitrile, purged with N_2_, then immediately stored in refrigerator at 4 °C for 12 h to gain preassembled solution. Then, 50.0 mg vinyl-modified Fe_3_O_4_@mSiO_2_, 4.0 mmol EGDMA and 20.0 mg AIBN were dissolved in 15.0 mL acetonitrile and added into aforementioned preassembled solution, purged with N_2_ on ice, then immediately allowed to react at 60 °C for 24 h under continuous stirring [37]. After polymerization, ending Fe_3_O_4_@mSiO_2_@MIPs were gathered magnetically, eluted with acetonitrile until the supernatant was transparent, and then rinsed with methanol-acetic acid (9/1, *v*/*v*) to remove the template completely, which affirmed with a UV-vis spectrometer at 238 nm (λ_max_ of cartap) [18]. In the end, the Fe_3_O_4_@mSiO_2_@MIPs were rinsed with methanol to neutralize pH and vacuum dried overnight at 50 °C. As a control, the same procedures were applied for the synthetization of Fe_3_O_4_@mSiO_2_@NIPs in the absences of template.

### 3.4. Adsorption Experiments

For adsorption equilibrium experiments, 10.0 mg Fe_3_O_4_@mSiO_2_@MIPs (or Fe_3_O_4_@mSiO_2_@NIPs) were suspended in a series of vials containing 3.0 mL cartap aqueous solutions with starting concentrations ranging from 0.1 to 25.0 mmol/L, respectively. The vials were shaken under 200 rpm for 3 h at 298 K, 308 K, and 318 K, respectively, and the equilibrium concentrations of cartap were analyzed by HPLC. The equilibrium adsorption capacity *Q*_e_ (mmol/g) was calculated by the following equation:

*Q*_e_ = (*c*_0_ − *c*_e_)*V*/*m* (3)

where *c*_0_ (mmol/L) and *c*_e_ (mmol/L) are the initial concentration and the equilibrium concentration of cartap, respectively. *V* (L) is the volume of cartap solution, while m is the mass of Fe_3_O_4_@mSiO_2_@MIPs or Fe_3_O_4_@mSiO_2_@NIPs (g).

For adsorption kinetic experiments, 50.0 mg Fe_3_O_4_@mSiO_2_@MIPs (or Fe_3_O_4_@mSiO_2_@NIPs) were suspended in a vial containing 15.0 mL cartap water solution (4.0 mmol/L). The vial was then continually shaken under 200 rpm at 298 K, and the concentrations of cartap in the supernatant at some intervals (5, 10, 20, 30, 40, 50, 60, 90, and 120 min) were analyzed by HPLC, and then the adsorption capacity *Q*_t_ (mmol/g) at different contact times t (min) was calculated as:

*Q*_t_ = (*c*_0_ − *c*_t_)*V*/*m* (4)

where *c*_t_ (mmol/L) is the concentration of cartap at different contact times.

Furthermore, adsorptive selectivity was executed using cartap in the presence of its four analogues including nereistoxin, bensultap, bisultap and monosultap (Figure 1) as standard mixed solutions with the initial concentrations of 4.0 mmol/L for each one. The imprinting factor *α* [45] was used to quantify the specific recognition of the Fe_3_O_4_@mSiO_2_@MIPs: *α* = *Q*_Fe__3O__4@mSiO__2@MIPs_/*Q*_Fe__3O__4@mSiO__2@NIPs_, where, *Q*_Fe__3O__4@mSiO__2@MIPs_ and *Q*_Fe__3O__4@mSiO__2@NIPs_ are the amount of cartap absorbed onto Fe_3_O_4_@mSiO_2_@MIPs and Fe_3_O_4_@mSiO_2_@NIPs, respectively. The separation factor *β* [45] was used to evaluate the selective recognition of Fe_3_O_4_@mSiO_2_@MIPs: *β* = *Q*_template_/*Q*_non-template_, where *Q*_template_ and *Q*_non-template_ were the adsorption amount of target molecules and control molecules on Fe_3_O_4_@mSiO_2_@MIPs, respectively.

### 3.5. Regeneration and Reused Experiments

Fe_3_O_4_@mSiO_2_@MIPs loaded with cartap from cartap reference solution were collected by a magnet, rinsed with acetonitrile to eliminate the nonspecific adsorption, and then eluted with methanol–acetic acid (9/1, *v*/*v*) (1.0 mL) for 1 h to reach complete cartap desorption. In addition, the Fe_3_O_4_@mSiO_2_@MIPs were washed with methanol to neutral pH, washed with distilled water for 3 times, and vacuum dried overnight at 50 °C to regenerate the Fe_3_O_4_@mSiO_2_@MIPs [46]. Then the regenerated Fe_3_O_4_@mSiO_2_@MIPs could be reused to identify reusability.

### 3.6. Selective Recognition of Cartap from Tea Beverages by Fe_3_O_4_@mSiO_2_@MIPs

After being oven-dried for 24 h at 50 °C, various tea products were ground to 40-mesh size. The ground samples were re-dried for 8 h at 50 °C. All six types of tea products and two tea beverages were firstly determined to be free of cartap by HPLC (with LOD of 1.0 × 10^−4^ mg/L) before use. Thus, 1.000 g tea products spiked with 1 mg/mL cartap (10 µL) were suspended in 25 mL deionized water of 90 °C. The suspensions were incubated at 90 °C for more 20 min before filtrating through 0.45 µm filter membrane to save the filtrates for analysis.

In addition, 3 mL of bottled tea beverages were spiked with different amounts of cartap (i.e., 0, 0.01, 0.05, 0.1, 0.5, 1.0, 5.0, 10, 20, and 30 mg/L), respectively, and then were used to analyze after filtrating through 0.45 µm filter membrane.

After that, Fe_3_O_4_@mSiO_2_@MIPs (10.0 mg) were suspended in the above spiked tea solution samples (3.0 mL). After shaking for 180 min under 200 rpm, Fe_3_O_4_@mSiO_2_@MIPs were gathered by a magnet, and then rinsed with acetonitrile and methanol-acetic acid (9/1, *v*/*v*) (1.0 mL) in turn at 20 °C to enrich cartap. The eluate was dried with N_2_, and the residue was dissolved in water and analyzed by HPLC and colorimetric/UV–vis spectroscopic methods.

### 3.7. Colorimetric, UV-Vis Spectroscopic and HPLC Determination of Cartap

AgNPs were synthesized by a simple chemical reduction method. In short, 4.0 mL of silver nitrate aqueous solution (0.5 mmol/L) was dropwisely added into 10.0 mL of freshly prepared sodium borohydride solution (2.0 mmol/L) under vigorous stirring at 300 rpm for 20 min at room temperature until the color of the solution turned to yellow [18]. The newly prepared AgNPs were applied to the following experiments right away (within 2 h).

The standard stock solutions of cartap at various concentrations (i.e., 0, 0.01, 0.05, 0.1, 0.5, 1.0, 5.0, 10, 20, and 30 mg/L) were prepared by dissolving cartap in deionized water. Typically, 100 µL of cartap water solution was added into 400 µL of newly synthesized AgNP solution. After gently shaking the mixtures for 10 min at room temperature, the color changes were observed by naked eyes and also the UV–vis absorption spectra (200–800 nm) were scanned and recorded [18]. The same processes were also applied for the aforementioned tea solution samples spiked with cartap, which were extracted with Fe_3_O_4_@mSiO_2_@MIPs.

The UV–vis absorption spectra of different concentrations of cartap standard solutions and spiked tea solutions extracted with Fe_3_O_4_@mSiO_2_@MIPs from three trials were averaged. The colorimetric detection of cartap in aqueous solution was operated at room temperature. The average absorbance values of all the concentrations or selected concentrations (i.e., 0.01, 0.1, 0.05, 1.0, and 5.0 mg/L) at 392 nm were plotted.

### 3.8. Statistical Analyses

All statistical analyses were performed using SPSS Statistics (v. 20, IBM, Armonk, NY, USA). Differences between the proposed method and traditional HPLC method were analyzed using one-way analysis of variance (ANOVA).

## 4. Conclusions

In summary, we have successfully established an AgNP colorimetric method with Fe_3_O_4_@mSiO_2_@MIPs as recognition elements to pretreat samples for measurement of cartap from tea beverages. The prepared Fe_3_O_4_@mSiO_2_@MIPs can effectively solidly extract and separate cartap in tea beverages, while AgNP colorimetry is able to determinate cartap. AgNP colorimetric sensor after Fe_3_O_4_@mSiO_2_@MIPs pretreatment is a rapid high-throughput method to easily identify high concentration of cartap by unaided eyes and is a quantificational mean to detect low concentration of cartap using a UV-vis spectrometer. In conclusion, this method exhibits great potential for rapid (the detection time with sample pretreatment <60 min) and accurate measurement of cartap in tea beverages demanded by the food supervision bureau and the food industry.

## Figures and Tables

**Table 1:** AgNP colorimetric detection after Fe_3_O_4_@mSiO_2_@MIPs-pretreatment and HPLC analysis of cartap in cartap-spiked green tea, black tea, white tea, yellow tea, dark tea, oolong tea, bottled green tea, and bottled iced black tea.

Samples *	AgNP Colorimetric Detection after Fe_3_O_4_@mSiO_2_@MIPs-Pretreatment	HPLC Analysis
Green tea (mg/kg)	10.112 ± 0.202 ^a^	10.084 ± 0.307 ^a^
Black tea (mg/kg)	9.798 ± 0.219 ^a^	9.831 ± 0.196 ^a^
White tea (mg/kg)	9.965 ± 0.199 ^a^	10.022 ± 0.287 ^a^
Yellow tea (mg/kg)	9.767 ± 0.235 ^b^	9.779 ± 0.109 ^b^
Dark tea (mg/kg)	10.033 ± 0.201 ^a^	9.974 ± 0.214 ^a^
Oolong tea (mg/kg)	10.042 ± 0.318 ^b^	10.013 ± 0.226 ^b^
Bottled green tea (mg/L)	4.027 ± 0.082 ^a^	4.008 ± 0.105 ^a^
Bottled iced black tea (mg/L)	3.985 ± 0.095 ^a^	4.015 ± 0.083 ^a^

* Each one gram of green tea, black tea, white tea, yellow tea, dark tea, oolong tea was spiked with 10 µL of cartap (1 mg/mL) before extracted with water, respectively; bottled green tea and bottled iced black tea were spiked with cartap till to 4 mg/L. The concentration of cartap was detected by UV-vis spectrometer with the AgNPs as colorimetric sensors. The results were shown on average value ± SD (*n* = 3). ^a^ Significant (two-tailed) at *p* ≤ 0.05 level. ^b^ Significant (two-tailed) at *p* ≤ 0.01 level.

## Supplementary Materials

The following are available online, Figure S1: The FT-IR spectra of (a) Fe_3_O_4_, (b) Fe_3_O_4_@CTAB/SiO_2_, (c) vinyl modified Fe_3_O_4_@mSiO_2_ and (d) Fe_3_O_4_@mSiO_2_@MIPs, Figure S2: Adsorption isotherms of cartap on (A) Fe_3_O_4_@mSiO_2_@MIPs and on (B) Fe_3_O_4_@mSiO_2_@NIPs, Figure S3: Adsorption kinetics curve of 4.0 mmol/L cartap on Fe_3_O_4_@mSiO_2_@MIPs and Fe_3_O_4_@mSiO_2_@NIPs at 318 K, Figure S4: Reusability of Fe_3_O_4_@mSiO_2_@MIPs, and Table S1: Adsorption isotherm constants for the Langmuir and Freundlich equations using adsorption data of cartap on Fe_3_O_4_@mSiO_2_@MIPs and Fe_3_O_4_@mSiO_2_@NIPs.
