# Supplementary material for: Rapid Colorimetric Detection of Cartap Residues by AgNP Sensor with Magnetic Molecularly Imprinted Microspheres as Recognition Elements
Source: Molecules. 2018 Jun 14;23(6):1443. doi: 10.3390/molecules23061443 (PMC6099834; doi:10.3390/molecules23061443)
Supplement: Supplementary file 1 [file molecules-23-01443-s001.pdf]

Supplementary Material

Rapidly Colorimetric Detection of Cartap Residues by AgNPs Sensor with Magnetic Molecularly Imprinted Microspheres as Recognition Elements

Mao Wu [1], Huiyun Deng [1], Yajun Fan [1], Yunchu Hu [1], Yaping Guo [1,*], and Lianwu Xie [1,2,*]

[1] College of Sciences, Central South University of Forestry and Technology, Changsha 410004, P. R. China
[2] College of Chemistry and Chemical Engineering, Central South University, Changsha 410083, P. R. China
* Correspondence: guoyaping@csuft.edu.cn (Y. Guo); xielianwu@csuft.edu.cn (L. Xie); Tel.: +86-7318-5623-648

Figure S1. The FT-IR spectra of (a) Fe_3O_4, (b) Fe_3O_4@CTAB/SiO_2, (c) vinyl modified Fe_3O_4@mSiO_2 and (d) Fe_3O_4@mSiO_2@MIPs.

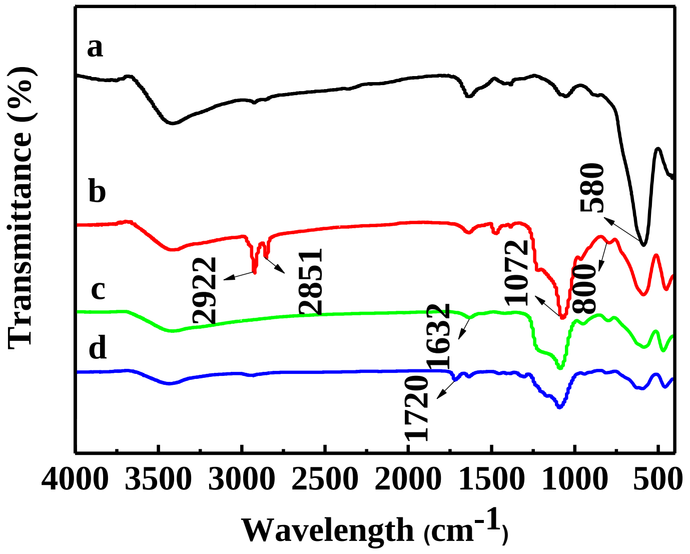

Figure S2. Adsorption isotherms of cartap on (A) Fe_3O_4@mSiO_2@MIPs and on (B) Fe_3O_4@mSiO_2@NIPs

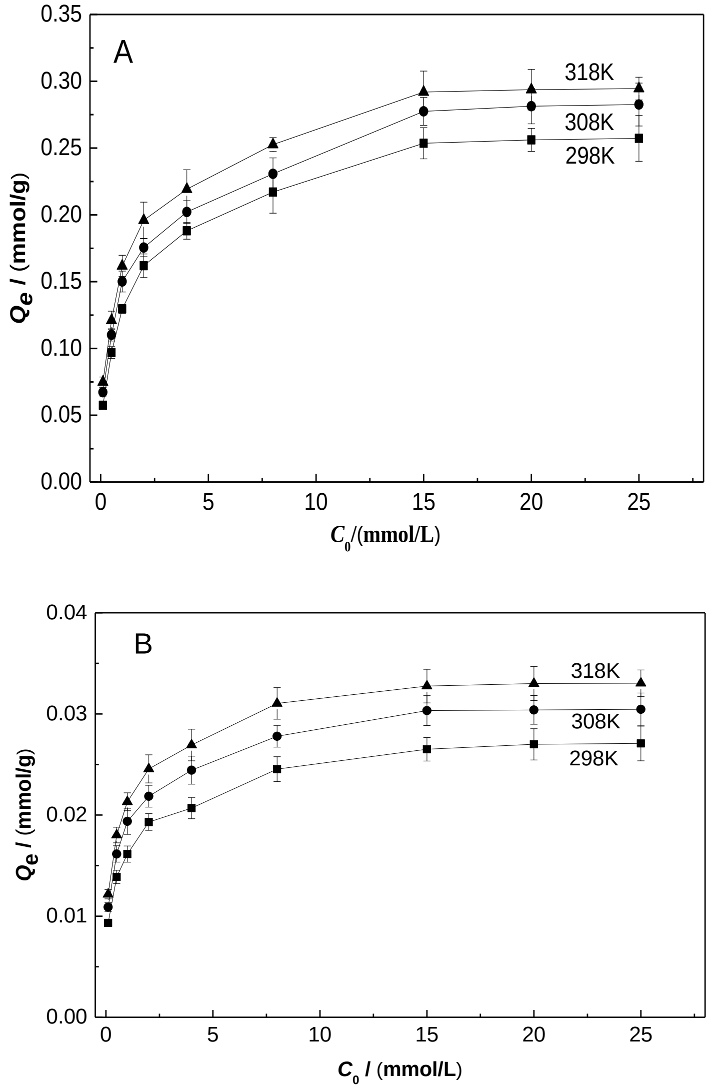

Figure S3. Adsorption kinetics curve of 4.0 mmol/L cartap on Fe_3O_4@mSiO_2@MIPs and Fe_3O_4@mSiO_2@MIPs at 318 K

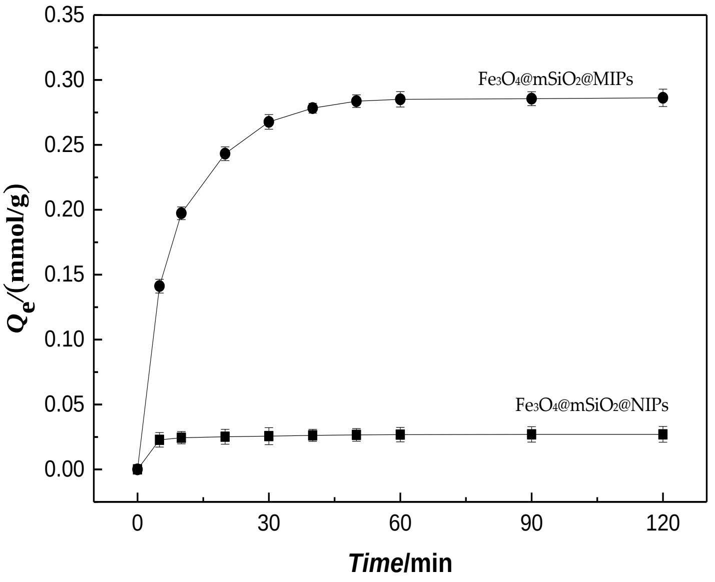

Figure S4. Reusability of Fe_3O_4@mSiO_2@MIPs.

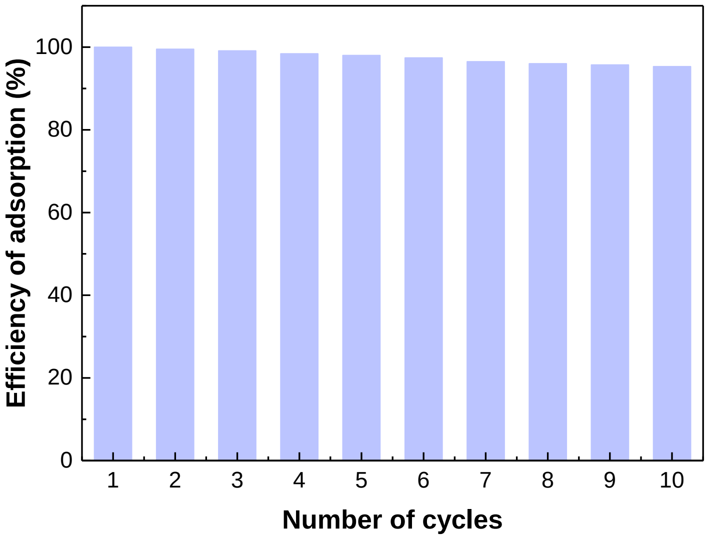

Table S1. Adsorption isotherm constants for the Langmuir and Freundlich equations using adsorption data of cartap on Fe_3O_4@mSiO_2@MIPs and Fe_3O_4@mSiO_2@NIPs.

T(K)		Langmuir model			Freundlich model		
		Q_m(cal) /(mmol/g)	K_L /(L/mmol)	R^2	n	K_f	R^2
298	Fe_3O_4@mSiO_2@MIPs	0.2509	2.2301	0.9966	2.4401	0.1387	0.8891
	Fe_3O_4@mSiO_2@NIPs	0.0218	4.6560	0.9964	3.4637	0.0159	0.8887
308	Fe_3O_4@mSiO_2@MIPs	0.2776	2.4952	0.9983	2.5724	0.1537	0.8884
	Fe_3O_4@mSiO_2@NIPs	0.0236	4.8673	0.9960	3.6294	0.0182	0.8723
318	Fe_3O_4@mSiO_2@MIPs	0.2867	2.6975	0.9952	2.7121	0.1644	0.8784
	Fe_3O_4@mSiO_2@NIPs	0.0252	5.0456	0.9975	3.8097	0.0199	0.8923
